# Supplementary material for: A Comprehensive Assessment of Informal Caregivers of Patients in a Primary Healthcare Home-Care Program
Source: Int J Environ Res Public Health. 2021 Nov 4;18(21):11588. doi: 10.3390/ijerph182111588 (PMC8583293; doi:10.3390/ijerph182111588)
Supplement: Supplementary file 1 [file ijerph-18-11588-s001.zip › ijerph-1440782-supplementary.pdf]

Supplementary Materials

**Health-related quality of life. Physical summary.**	**β**	**SE**	**95% CI**		**p-value**
Social support status					
**Social problem or at social risk**	-2,03	2,45	-6,82	2,77	0,41
**Social Support (Duke)**	0,07	0,12	-0,16	0,30	0,56
**Social Class**					
I and II (Higher managerial, administrative, and professional occupations and lower managerial)			*ref*		
III (Intermediate occupations)	2,47	3,83	-5,03	9,97	0,52
IV (Small employers and own-account workers)	-2,19	4,17	-10,37	5,98	0,60
V (Lower supervisory and technical occupations)	0,86	3,25	-5,52	7,24	0,79
VI (Semi-routine occupations)	-4,55	3,83	-12,04	2,95	0,23
**Shared care**					
No shared care			*ref*		
Shared with another informal caregiver	0,52	2,65	-4,67	5,70	0,85
Shared with a formal caregiver	2,43	2,79	-3,04	7,89	0,38
**Time of care in hours weekly**	*-0,05*	*0,10*	*-0,25*	*0,15*	*0,01*
**The patient lives at home of the carer**	*-5,98*	*3,23*	*-12,30*	*0,35*	*0,06*
**Number of residents at home**	1,33	1,19	-1,01	3,66	0,27
Clinical Characteristics					
**Sex (male)**	*-1,19*	*2,71*	*-6,50*	*4,13*	0,66
**Age**	*-0,25*	*0,08*	*-0,41*	*-0,73*	*0,003*
**Depresion**	0,29	2,53	-4,66	-6,62	0,91
**COPD**	*-8,48*	*2,96*	*-14,27*	*-25,02*	*0,004*
**Back pain**	-2,88	2,25	-7,30	-12,05	0,20
**High Hipertension**	*-6,39*	*2,04*	*-10,40*	*-18,33*	*0,002*
**Diabetis Mellitus**	*-7,18*	*2,47*	*-12,01*	*-21,08*	*0,004*
**Dislipemia**	-2,42	2,14	-6,62	-10,84	0,26
**Alcohol problems**	-0,31	4,90	-9,92	-14,53	0,95
**Cardiovascular disease**	-1,30	3,07	-7,31	-11,26	0,67
**Commorbility (Weighted Charlson Index)**					
No commorbility			*ref*		
Low commorbility	-2,00	3,17	-8,20	-12,91	0,53
High commorbility	*-6,63*	*2,67*	*-11,86*	*-20,58*	*0,01*
Factors from the patient.					
**Age**	-0,07	0,16	-0,39	0,24	0,64
**Sex (male)**	1,101	2,233	-3,276	5,478	0,623
**Dependency (Barthel Index)**					
Moderate dependency			*ref*		
Severe dependecy	*-5,77*	*2,87*	*-11,39*	*-19,46*	*0,04*
Total dependency	*-7,35*	*3,01*	*-13,26*	*-22,97*	*0,01*
**FIM Motor**	0,05	0,05	*-0,04*	*-0,03*	0,25
**FIM Cognitive**	-0,16	0,12	-0,39	0,07	0,18

Table SI: Bivariate regression models (Mod1) Health-related quality of life. Physical summary.

Health-related quality of life. Mental summary.	β	SE	95% CI		p-value
Social support status					
**Social problem or at social risk**	-4,47	2,68	-9,71	0,78	0,10
**Social Support (Duke)**	0,48	0,11	0,26	0,70	<0.001
**Social Class**					
I and II (Higher managerial, administrative, and professional occupations and lower managerial)			ref		
III (Intermediate occupations)	1,20	4,30	-7,23	9,62	0,78
IV (Small employers and own-account workers)	-2,31	4,69	-11,50	6,87	0,62
V (Lower supervisory and technical occupations)	0,27	3,66	-6,90	7,43	0,94
VI (Semi-routine occupations)	3,05	4,30	-5,38	11,48	0,48
**Shared care**					
No shared care			ref		
Shared with another informal caregiver	0,16	2,94	-5,59	5,91	0,96
Shared with a formal caregiver	1,45	3,09	-4,61	7,51	0,64
**Time of care in hours weekly**	-0,03	0,02	-0,07	0,01	0,21
**The patient lives at home of the carer**	0,90	3,63	-6,22	8,01	0,81
**Number of residents at home**	-1,12	1,32	-3,71	1,46	0,39
Clinical Characteristics					
**Sex (male)**	9,68	2,84	4,10	15,25	0,001
**Age**	0,29	0,092	0,11	0,31	0,002
**Depresion**	-11,44	2,55	-16,45	-29,68	<0.001
**COPD**	-4,84	3,36	-11,43	-19,04	0,15
**Back pain**	-7,10	2,41	-11,83	-20,77	0,00
**High Hipertension**	0,86	2,36	-3,77	-5,03	0,72
**Diabetis Mellitus**	0,31	2,84	-5,26	-7,47	0,91
**Dislipemia**	0,68	2,38	-3,99	-5,43	0,77
**Alcohol problems**	-10,85	5,31	-21,26	-36,36	0,04
**Cardiovascular disease**	4,52	3,37	-2,09	-0,72	0,18
**Commorbility (Weighted Charlson Index)**					
No commorbility			ref		
Low commorbility	0,03	3,57	-6,96	-10,07	0,99
High commorbility	4,59	3,01	-1,30	0,46	0,13
Factors from the patient.					
**Age**	0,23	0,18	-0,12	0,58	0,20
**Sex (male)**	1,26	2,47	-3,58	6,10	0,61
**Dependency (Barthel Index)**					
Moderate dependency			ref		
Severe dependecy	4,17	3,24	-2,18	-1,03	0,20
Total dependency	3,13	3,40	-3,54	-3,54	0,36
**FIM Motor**	0,03	0,05	-0,07	-0,09	0,55
**FIM Cognitive**	0,13	0,13	-0,13	0,38	0,33

Table SII: Bivariate regression models (Mod1) Health-related quality of life. Mental summary.

**Zarit burden interview associated factors..**	**β**	**SE**	**95% CI**		**p-value**
		Social support status			
**Social problem or at social risk**	4,71	3,93	-3,00	12,41	0,23
**Social Support (Duke)**	-0,69	0,15	-0,99	-0,39	<0.001
**Social Class**					
I and II (Higher managerial, administrative, and professional occupations and lower managerial)			ref		
III (Intermediate occupations)	2,96	6,16	-9,12	15,03	0,63
IV (Small employers and own-account workers)	10,26	6,72	-2,90	23,43	0,13
V (Lower supervisory and technical occupations)	7,06	5,24	-3,20	17,33	0,18
VI (Semi-routine occupations)	-0,75	6,16	-12,82	11,33	0,90
**Shared care**					
No shared care			Ref		
Shared with another informal caregiver	-3,36	4,27	-11,73	5,02	0,43
Shared with a formal caregiver	-2,75	4,50	-11,57	6,07	0,54
**Time of care in hours weekly**	0,01	0,03	-0,06	0,08	0,76
**The patient lives at home of the carer**	0,50	5,29	-9,88	10,87	0,93
**Number of residents at home**	3,75	1,90	0,03	7,46	0,05
		Clinical Characteristics			
**Sex (male)**	-3,74	4,36	-12,28	4,81	0,39
**Age**	-0,40	0,13	-0,66	-1,17	0,00
**Depresion**	12,43	3,88	4,81	13,32	0,00
**COPD**	10,20	4,85	0,69	6,20	0,04
**Back pain**	7,20	3,59	0,16	3,90	0,05
**High Hipertension**	-3,29	3,43	-10,02	-16,20	0,34
**Diabetis Mellitus**	-3,07	4,13	-11,17	-17,76	0,46
**Dislipemia**	-7,12	3,40	-13,79	-23,63	0,04
**Alcohol problems**	-3,89	7,90	-19,37	-30,06	0,62
**Cardiovascular disease**	-3,84	4,94	-13,52	-21,56	0,44
**Commorbility (Weighted Charlson Index)**					
No commorbility			Ref		
Low commorbility	-7,78	5,12	-17,81	-29,79	0,13
High commorbility	-11,63	4,31	-20,09	-35,06	0,01
		Factors from the patient.			
**Age**	-0,25	0,26	-0,76	0,26	0,33
**Sex (male)**	3,70	3,59	-3,33	10,73	0,31
**Dependency (Barthel Index)**					
Moderate dependency			Ref		
Severe dependecy	-5,76	4,73	-15,03	-24,74	0,23
Total dependency	-4,53	4,97	-14,26	-22,99	0,36
**FIM Motor**	-0,03	0,08	-0,18	-0,27	0,71
**FIM Cognitive**	-0,24	0,19	-0,61	0,13	0,21

Table SIII: Bivariate regression models (Mod1) Zarit burden interview associated factors.

Backward stepwise models selection with the Akaike criterion (AIC) (Last step and final model).

Health-related quality of life. Physical summary.

```
Step:  AIC=770.71
Physical summary ~ TIME OF CARE + C.RESPIRATORY DISEASE + HYPERTENSION + HC_DM +
BARTHEL

                Df Deviance    AIC
<none>               8633.1 770.71
- TIME OF CARE   1   8890.3 771.77
- DIABETES M.          1   8910.2 772.00
- HYPERTENSION   1   8910.5 772.00
- C.RESP DISEASE 1   9040.5 773.51
- BARTHEL    2   9388.5 775.44

Call:  glm(formula = Physical summary ~ TIME OF CARE  + C.RESPIRATORY DISEASE  +
HYPERTENSION + DIABETES M. + BARTHEL, family = "gaussian", data = Data)

Coefficients:
(Intercept)  TIME OF CARE     C.RESPIRATORY DISEASE   HYPERTENSION1  DIABETES M.1
 56.36530      -0.03354             -5.95737                -3.97273     -4.63555

BARTHEL2   BARTHEL3
-6.32993   -8.01545

Degrees of Freedom: 103 Total (i.e. Null);  97 Residual
Null Deviance:        11660
Residual Deviance: 8633      AIC: 770.7
```

Health-related quality of life. Mental summary.

```
Start:  AIC=761.31
MENTAL summary ~ DUKE + SEX + AGE + DEPRESION + BACK PAIN

                   Df Deviance    AIC
<none>                  8039.7 761.31
- BACK PAIN         1   8317.1 762.83
- AGE               1   8429.4 764.23
- DEPRESION         1   8715.5 767.70
- SEX               1   9438.9 775.99
- DUKE              1   9523.4 776.92

Call:  glm(formula = MENTAL SUMMARY ~ DUKE + SEXO + EDAD_N_CUIDADOR + DEPRESION +
    BACK PAIN, family = "gaussian", data = Data)

Coefficients:
 Intercept)        DUKE      SEX1       AGE     DEPRESION1    BACK PAIN1
  14.0060        0.4152  10.0200    0.1656    -6.5185      -3.6756
```

```
Degrees of Freedom: 103 Total (i.e. Null);  98 Residual
Null Deviance:      14270
Residual Deviance: 8040      AIC: 761.3
```

Zarit burden interview

```
Step:  AIC=861.32
ZARIT~DUKE + N_HOUSEHOLD RESIDENTS + DEPRESION + C.RESPIRATORY DISEASE + DYSLIPIDEMIA

                            Df Deviance    AIC
<none>                            21032 861.32
- C.RESPIRATORY DISEASE      1    21624 862.21
- DYSLIPIDEMIA               1    21866 863.36
- DEPRESION                  1    22279 865.31
- N_HOUSEHOLD RESIDENTS      1    22303 865.42
- DUKE                       1    24289 874.29

Call:  glm(formula = ZARIT ~ DUKE + N_HOUSEHOLD RESIDENTS+ DEPRESION +
    C.RESPIRATORY DISEASE  + DYSLIPIDEMIA , family = "gaussian", data = Data)

Coefficients:
 Intercept)    DUKE     N_HOUSEHOLD RESIDENTS      HC_DEPRESION1
   47.3336    -0.5834           4.0528                 8.6726

C.RESPIRATORY DISEASE 1    DYSLIPIDEMIA 1
  7.1078                    -5.9148

Degrees of Freedom: 103 Total (i.e. Null);  98 Residual
Null Deviance:     30340
Residual Deviance: 21030     AIC: 861.3
```
